# lncRNAs: function and mechanism in cartilage development, degeneration, and regeneration

**DOI:** 10.1186/s13287-019-1458-8

**Published:** 2019-11-21

**Authors:** Jian Zhu, Wei Yu, Yitian Wang, Kaishun Xia, Yuluan Huang, Ankai Xu, Qixin Chen, Bing Liu, Huimin Tao, Fangcai Li, Chengzhen Liang

**Affiliations:** 1 Department of Orthopedics, 2nd Affiliated Hospital, School of Medicine, Zhejiang University, #88 Jie Fang Road, Hangzhou, 310009 Zhejiang People’s Republic of China; 2 Orthopedics Research Institute of Zhejiang University, #88, Jiefang Road, Hangzhou, 310009 China; 3 Department of Gynecologic Oncology, Women’s Hospital, School of Medicine, Zhejiang University, Hangzhou, 310009 China

**Keywords:** LncRNA, Cartilage, Arthritis, IDD, Mesenchymal stem cell, Differentiation

## Abstract

With the increasing incidence of cartilage-related diseases such as osteoarthritis (OA) and intervertebral disc degeneration (IDD), heavier financial and social burdens need to be faced. Unfortunately, there is no satisfactory clinical method to target the pathophysiology of cartilage-related diseases. Many gene expressions, signaling pathways, and biomechanical dysregulations were involved in cartilage development, degeneration, and regeneration. However, the underlying mechanism was not clearly understood. Recently, lots of long non-coding RNAs (lncRNAs) were identified in the biological processes, including cartilage development, degeneration, and regeneration. It is clear that lncRNAs were important in regulating gene expression and maintaining chondrocyte phenotypes and homeostasis. In this review, we summarize the recent researches studying lncRNAs’ expression and function in cartilage development, degeneration, and regeneration and illustrate the potential mechanism of how they act in the pathologic process. With continued efforts, regulating lncRNA expression in the cartilage regeneration may be a promising biological treatment approach.

## Introduction

Nowadays, the disease owing to cartilage widely influences people’s lives, especially in aging society countries. Cartilage diseases such as osteoarthritis (OA) and intervertebral disc degeneration (IDD) will cause pain and movement limitations [1]. The main cause of OA and IDD is the progressive destruction of cartilage [2, 3]. In order to cure osteoarthritis and intervertebral disc degeneration, it is necessary to understand the cartilage’s development, degeneration, and regeneration.

LncRNAs attract more and more attention owing to their abundance functions in various tissues [4]. LncRNAs are virtually transcribed by RNA polymerase II and contain RNA-processing signals such as poly (A) tails and 5′ caps [5]. Owing to the lack of open reading frame, lncRNAs were thought of as “junk RNAs.” On the progress in the research, lncRNAs were found to act a crucial role in the biological process. LncRNAs are alternatively spliced and undergo a process to remove the intronic sequence [6]. LncRNAs are about 200 nucleotides to 100 kb, similar in the structure of mRNA transcripts but without encoding a protein function [7]. According to the location relative to the gene locus, lncRNAs can be divided into five categories: sense, antisense, bidirectional, intronic, and intergenic [8]. LncRNAs are more species-specific and less conserved than the protein-encoding genes. LncRNAs can act as a regulator in various biological processes such as tumor development, stem cell differentiation, epigenetic regulation, immune response, and inflammation-related diseases [9–13]. LncRNAs can act as an indicator, biomarker, and therapy target in the physiologic and pathologic processes, including cartilage development, degeneration, and regeneration [14–16].

Now in this report, we will illustrate the role of lncRNAs in cartilage development, degeneration, and regeneration [14, 17] (Table 1). Hopefully, the brief introduction could afford a deep understanding of chondrocyte degeneration and a new target to cure cartilage degeneration.

**Table 1:** Functional characterization of lncRNAs in text

LncRNAs	Expression	Functional role	Related factor	Reference
lncRNA-CIR	Up	Aggrecan and collagen degradation	MMP-13 and ADAMTS5	[15]
RP11-296A18.3	Up	Abnormal proliferation of HNPC	miR-138 and HIF1α	[18]
PART1	Up	Influence the expression of ACVR1, E2F3, and VEGFA	miR-34a and miR-148a	[19]
HOTAIR	Up	Overexpression of matrix metalloproteinase	IL-1β	[20]
CILinc01 and CILinc02	Down	Influence cytokine production	NF-κB	[21]
PACER	Up	Influence cytokine production	COX-2	[22]
AC005082.12	Up	ECM degeneration	EFNA3	[23]
MEG3	Down	Vascular invasion	VEGF	[24]
HCG18	Up	NP cell apoptosis	miR-146a-5p/TRAF6/NFκB	[25]
HOTTIP	Up	Chondrogenic differentiation inhibition	HoxA13	[26]
GAS5	Up	Apoptosis of chondrocytes	miR-21	[27]
PCGEM1	Down	Apoptosis of synoviocyte	miR-770	[28]
linc-ADAMTS5	Down	Aggrecan degradation	ADAMTS5	[29]
TUG1	Up	NP cell proliferation inhibition	Wnt β-catenin	[30]
LncRNA-MSR	Up	Overexpression of metalloproteinase	miR-152	[31]
DANCR	Down	Chondrogenic differentiation inhibition	smad3 and STAT3	[32]

## The role of lncRNAs in cartilage development

**Fig. 1:**
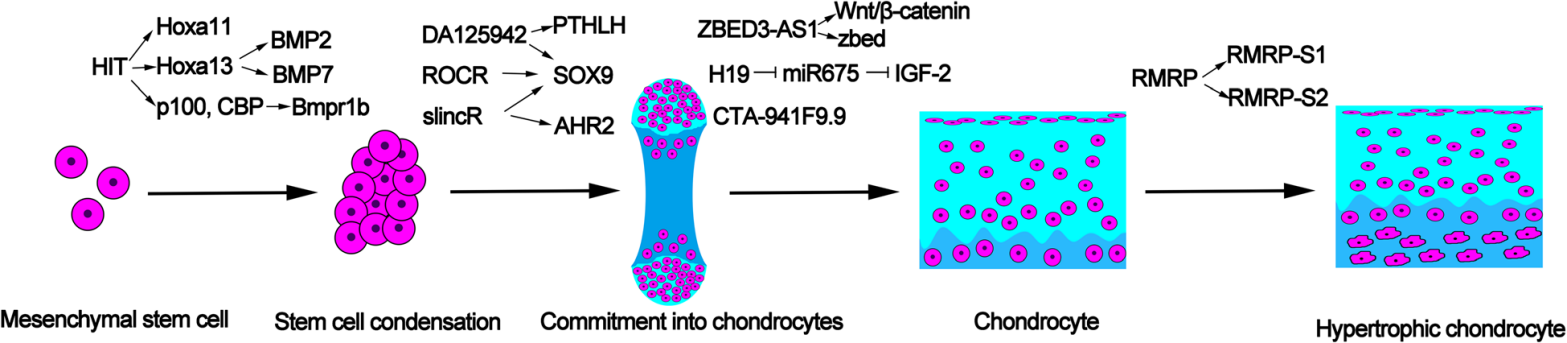
LncRNA HIT regulates mesenchymal stem cells through LncRNA DA125942, ROCR, and slincR which influence the expression of SOX9 which is important in the early stage of chondrocyte differentiation. LncRNA ZBED-AS1, H19, and CTA-941F9.9 are involved in the process of chondrocyte differentiation. LncRNA RMRP could promote the chondrocyte differentiating to hypertrophic chondrocytes

Cartilage could be divided into three types: hyaline, elastic, and fibrocartilage [33]. Cartilage was thought of as a simple structure, because it contains only one type of cell and its extracellular matrix (ECM) contains only three components: water, collagen, and proteoglycan [34]. Chondrogenesis is the primary process in cartilage development [35]. Cartilage development contains five stages [36]: the initial stage mesenchymal stem cell, commitment into chondrocyte, chondrocyte differentiation, chondrocyte hypertrophy, and calcification and degradation of cartilage matrices (Fig. 1).

In the initial stage of cartilage formation, mesenchyme cells begin to condense. The BMP-SMAD4 signaling pathway plays a crucial role in mesenchymal condensation [37]. BMPR1B is a principal receptor for BMPs and GDF5. It will induce the skeletal element hypoplasia for the lack of BMPR1B. LncRNA HIT could regulate the BMP-SMAD4 signaling pathway through two mechanisms [38]. Firstly, LncRNA HIT binds to its associated protein such as p100 and CBP to regulate the expression of Bmpr1b. Secondly, lncRNA HIT locates within the HOXA locus [39]. LncRNA HIT could regulate the expression of Hoxa13 and Hoxa11. Meantime, Hoxa13 regulates Bmp2 and Bmp7, and Hoxa11 regulates Runx2 in the BMP signaling pathway. Runx2 was reported to be expressed in cartilage condensation in the cartilage anlagen of the forelimb zeugopod [40]. Therefore, lncRNA HIT plays a major role in the initial stage of cartilage formation.

Sex-determining region Y (SRY)-box 9 (Sox9) plays an important role in promoting mesenchymal stem cells to the stage of commitment into chondrocytes [41]. Sox9 determines cell fate in cells derived from all three germ layers. It plays an important role in the initial stage of cartilage development. Mutation of Sox9 will disrupt the cartilage formation to cause campomelic dysplasia [36]. Aryl hydrocarbon receptor (AHR) is a conserved receptor from invertebrates to vertebrates, loss of which will protect against toxicity phenotypes, including cardiac malformation, cartilage malformation, and reduced peripheral blood flow [42, 43]. Garcia et al. [44] found that a novel lncRNA named slincR is associated with AHR2 and sox9b expression during normal development. LncRNA slincR acts as an intermediate between AHR2 and sox9b mRNA. However, more researches are needed to illustrate the mechanism of that reduction of sox9b caused by LncRNA slincR. Another lncRNA termed lncRNA ROCR was located 94 kb upstream of the location of SOX9. LncRNA is prone to regulate the expression of the gene nearby [45]. LncRNA ROCR aggregates more in the cytoplasm than in the nucleus, suggesting that it may regulate the expression of SOX9 in an indirect way. RNAi and LNA GapmeR approach were used to identify the effect of lncRNA ROCR on the expression of SOX9 and chondrogenesis [46]. The study showed that lncRNA ROCR contributes to the expression of SOX9, and lncRNA ROCR is necessary for matrix GAG production. All the data support that lncRNA ROCR is important for chondrogenesis. LncRNA ROCR in the study was detected from RNA extracted from an aged neck of femur and OA tissue. Further work should confirm the expression of lncRNA ROCR in normal tissue. Morphogenesis gene PTHLH regulates cartilage differentiation and digit condensation and was involved in SOX-9-mediated chondrogenesis. CISTR-ACT that encodes a lncRNA termed as DA125942 was found to regulate PTHLH expression in *cis* and SOX-9 expression in *trans*. PTHLH and other factors influence the BMP-mediated and SOX9-directed chondrogenesis through balancing a complex signaling network. DA125942 could inhibit the expression of PTHLH and SOX9. Therefore, downregulating the expression of DA125942 may be an approach to promote chondrogenesis [47].

Emerging studies showed that lncRNAs play a part in the stage of chondrocyte progenitor cells into chondrocyte differentiation [48]. H19 is an imprinted maternally gene during fetal development [49]. And H19 may play a role by harboring the miR-675 [50]. Steck and his colleagues [51] have found that the H19-encoded miR-675 modulates collagen type II levels. Insulin growth factor (IGF) is from paternal and is a neighboring gene of H19. H19 influences IGF-2 through sponging miR-675. miR-675 could regulate the expression level of COL2A1 by SOX-9 [52]. In addition, the study revealed that anabolic stimuli upregulated the expression of H19/miR-675 while inflammatory cytokines downregulated them, and their overexpression may be good for cartilage anabolism and tissue degeneration. Meantime, two other lncRNA expression trends of ZBED3-AS1 and CTA-941F9.9 were observed during the chondrogenic differentiation process. Wang et al. [53] demonstrated that the two lncRNAs may function in the early stage of chondrogenic differentiation. Further studies by Ou et al. [54] found that ZBED3-AS1 could activate the Wnt/β-catenin signaling and increased the zbed expression. Overexpression of ZBED3-AS1 upregulates the expression levels of sox9 and collagen II, but the detailed mechanism requires further investigation.

The impaired cartilage development will cause cartilage-hair hypoplasia (CHH). Cartilage-hair hypoplasia is also termed metaphyseal chondrodysplasia. As a result, chondrocytes cannot develop into late phase/hypertrophic chondrocyte [55]. Sox-9 is expressed from the skeletogenetic progenitor cell to cartilage hypertrophy [56]. Mef2c and Runx2 function in the process from chondrocyte into hypertrophic chondrocyte [57]. Sox9 could be bind to the *cis*-element of Col2a1 to regulate chondrogenic differentiation while Runx2 and Mef2c regulate the expression of col10a1 during chondrogenic differentiation to the late phase. The mutation of the RMRP gene was reported as the main cause of CHH. RMRP lncRNA and some protein subunits form the small nucleolar ribonucleoprotein particle RNase MRP. RNase MRP is the source of two short RNA designated RMRP-S1 and RMRP-S2 [58]. Mutations in RNase MRP cause human cartilage-hair hypoplasia (CHH). During the course of chondrogenic differentiation, RMRP RNA was found to be involved in the chondrocyte hypertrophy while interfering RMPR RNA will lead to the deregulation of chondrogenic differentiation [59].

## Role of lncRNAs in cartilage degeneration

**Fig. 2:**
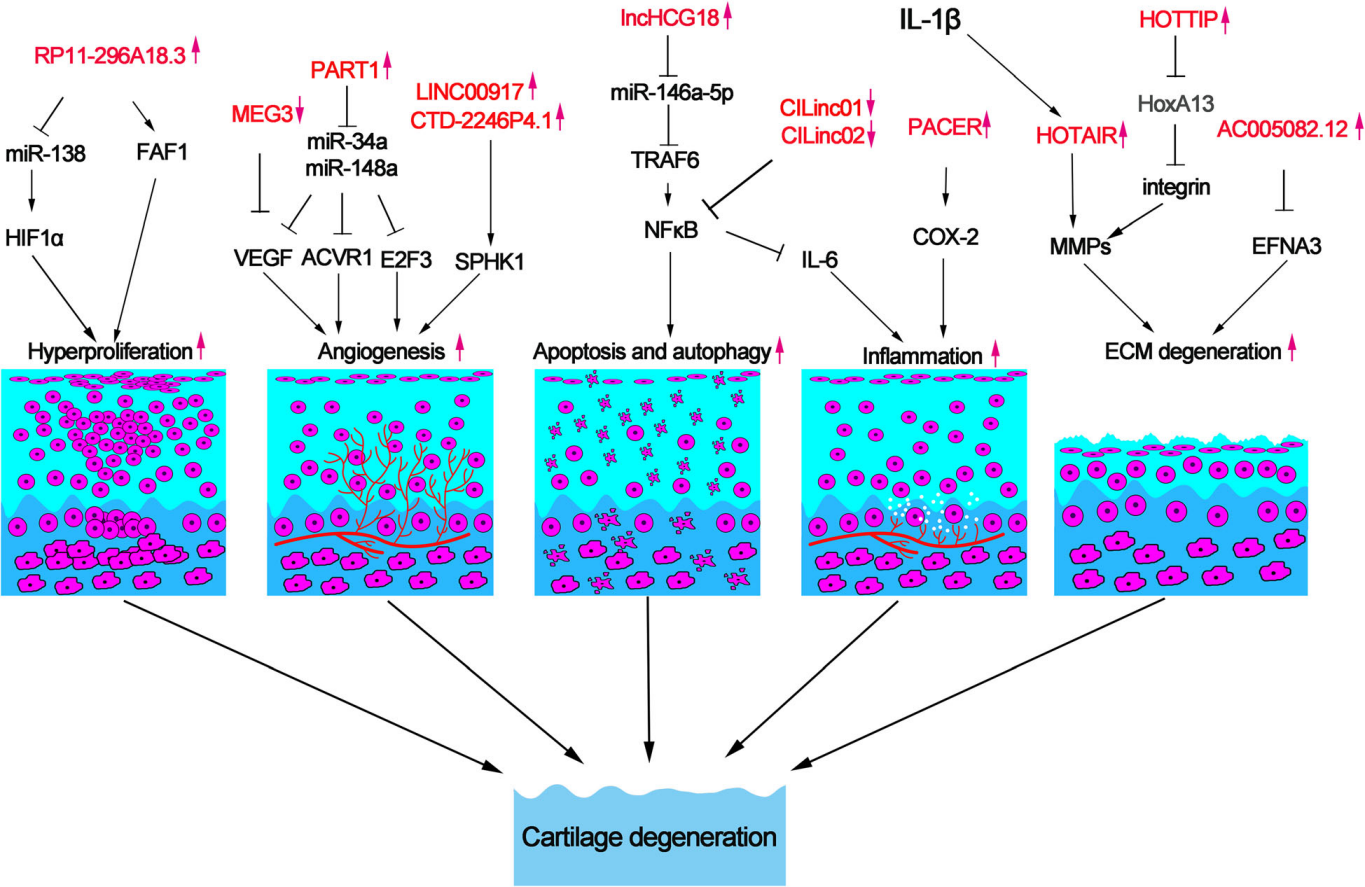
Inflammation, angiogenesis, hyperproliferation, ECM degeneration, apoptosis, and autophagy are the main causes of cartilage degeneration. LncRNA HOTAIR, PACER, CILinc01, and CILinc01 are involved in the inflammation process. LncRNA MEG3, PART1, LINC00917, and CTD-2246P4.1 promote the angiogenesis through regulating the expression of vascular factor. LncRNA RP11-296A18.3 acts as a sponge of miR-138 to induce chondrocytes hyperproliferation. LncHCG18 induces apoptosis and autophagy of chondrocyte through the NF-kB pathway. LncRNA HOTAIR, AC005082.12, and HOTTIP play a crucial role in the process of ECM degeneration

The degeneration of cartilage could lead to diseases such as osteoarthritis and intervertebral disc degeneration. These diseases induce pain and movement limitations and increase the social burden [60]. Diseases caused by cartilage degeneration were owing to inflammation, oxidative stress, angiogenesis, cell hyperproliferation, ECM degeneration, and chondrocyte apoptosis and autophagy [61–63] (Fig. 2). However, the underlying mechanism was elusive. In recent years, many lncRNAs were found to be correlated with osteoarthritis and intervertebral disc degeneration [17, 18]. A recent study identified lncRNAs in IDD and spinal cord injury as control with RNA sequencing (RNA-seq). In this study, 1854 lncRNAs were found differentially expressed, of which 1530 lncRNAs could influence 6386 genes through *cis*-regulatory mechanism [19]. A review described by Li shows that lncRNAs RP11-296A18.3, TUG1, HCG18, MALAT1, SNHG1, H19, NEAT1, and linc-ADAMTS5 were involved in the IDD process through regulating NP cells [64]. Another study showed that lncRNAs including Gm42770, XLOC-006055, Gm9801, RP23-54G8.4, Gm26848, A530020G20Rik, and Rian comprise the core regulatory network in OA [65].

Inflammation factors such as IL-1β and 6 and TNF play crucial roles in the development of osteoarthritis [66]. IL-1β could promote the brain-derived neurotrophic factor (BDNF) and vascular endothelial growth factor (VEGF) which induce angiogenesis [67]. IL-1β was also reported regulating cartilage catabolism, anabolism, and extracellular matrix synthesis [68]. Hox transcript antisense intergenic RNA (HOTAIR) was upregulated about 21-fold in OA compared to the normal cartilage tissue and reported to bind to polycomb repressive complex 2 (PRC2) [69]. Upregulation of IL-1β activated the expression of lncRNA HOTAIR which induced the overexpression of matrix metalloproteinase (MMP) family such as MMP1, MMP3, and MMP9 and chondrocytes apoptosis [20]. In addition, Inflammation factors could induce cartilage degeneration by regulating the expression of lncRNAs. Preculturing with IL-1β, 125 lincRNAs were detected differentially expressing in chondrocyte. The lincRNA p50-associated cyclooxygenase 2-extragenic RNA (PACER) and chondrocyte inflammation-associated lincRNAs (CILinc01 and CILinc02) were upregulated to influence the cytokine production, which play a crucial role in the inflammation-driven cartilage degeneration [21]. LincRNA PACER is located upstream to COX-2 locus and regulates COX-2 expression [22]. Silencing CILinc01 and CILinc02 could increase the expression of IL-6 via suppressing NF-kB’s activity. And the inhibitor IKK1 of the NF-kB pathway decreased the expression of CILinc01 and CILinc02. In conclusion, CILinc01 and CILinc02 could negatively regulate the inflammation factors to delay cartilage degeneration.

Meantime, angiogenesis is associated with the development of cartilage degeneration [70]. Degeneration of ECM will result in the migration of endothelial cells to cause neovascularization. Intervertebral disc is an avascular and immune-privileged organ [71]. The neovascularization will expose NP to the immune system to cause an immune response, which results in degeneration disease [72]. SPHK1 is a member of SPHK family, which is associated with cell migration and angiogenesis [73]. LncRNAs LINC00917 and CTD-2246P4.1 [23] were reported to play a crucial role in the development of IDD through influencing SPHK1 to regulate vascular generation. LncRNA PART1 influences the expression of ACVR1, E2F3, and VEGFA through interacting with has-miR-34a and has-miR-148a [19]. Although RNA-seq data were validated by qRT-PCR, more research should be done to explore the function and mechanism of LncRNA PART1. Maternally expressed gene 3 (MEG3) is located in chromosome 14q32 and acts as an inhibitor in tumor progress by inhibiting angiogenesis [74]. Moreover, lncRNA MEG3 that interacts with SOX2 influences the expression of BMP4 to promote osteogenic differentiation [75]. Meantime, angiogenesis and inflammation are causes of osteoarthritis [76]. LncRNA MEG3 is downregulated and inversely associated with VEGF expression, which causes cartilage remodeling and vascular invasion [24]. These findings suggest that lncRNA MEG3 may play a potential role in cartilage degeneration, although more work should be done to illustrate the underlying mechanism.

Human nucleus pulposus cells (HNPCs), small chondrocyte-like cells, are crucial in the homeostasis of the intervertebral disc. Abnormal proliferation of HNPC will generate cell clusters which cause intervertebral disc degeneration [77]. LncRNA-RP11-296A18.3 promotes the proliferation of HNPCs through sponging miR-138 which inhibits the expression of hypoxia-inducible factor-1α (HIF1α) [18]. HIF1α is an element that could lead to a massive death of HNPCs [78]. On the contrary, another study found that LncRNA-RP11-296A18.3 could promote the expression of Fas-associated protein factor-1 (FAF1), which induced aberrant apoptosis of cartilage cell through the Fas-mediated pathway [79]. Interestingly, LncRNA-RP11-296A18.3 could promote the proliferation of HNPCs but induce apoptosis of cartilage cells in different studies.

Chondrocyte autophagy and apoptosis play a crucial role in the development of cartilage degeneration [80]. Autophagy could remove the generation of reactive oxygen species (ROS) stimulated by a compression stimulus in the nucleus pulposus (NP) cells through sequestrating damaged organelles. In the study, SIRT1 could protect NP cells against apoptosis through promoting autophagy [81]. Interestingly, a recent study reported that the osteogenic differentiation of NP cells was associated with the development of IDD. In the study, Xi et.al found that lncHCG18 could activate the miR-146a-5p/TRAF6/NFκB axis which induced apoptosis and osteogenic differentiation of NP cells and macrophage recruitment [25]. LncHCG was highly expressed in IDD patient, so it may be used as an early diagnostic marker of IDD. Taken together, lncRNAs play a role in cartilage degeneration through inducing apoptosis and autophagy.

ECM serves as the culture medium for the chondrocytes and also serves as the bridge to transfer signals among different chondrocytes [82]. Collagens are the major components of the cartilage structure. Collagen-1 and MMP-13 are degeneration ECM markers [83]. Cartilage matrix protein binds to integrin to modulate processes in cartilage development and degeneration [84]. LncRNA CTC-523E23.5, RP4-639 J15.1, and RP11-363G2.4 were identified to interact with integrin [23]. So, knowing how to regulate integrin is important to study the cartilage degeneration. Dysregulation of HOX family transcripts may result in limb malformation [85]. HOTTIP, which is known as a regulator of the HoxA gene, was located at the 5′ end of HoxA cluster [86]. In the research studied by Kim et al., HOTTIP was found to regulate integrin by modulating HoxA13 [26]. Moreover, Chen et al. [23] found that lncRNA AC005082.12 interacts with Ephrin-A3 (EFNA3) while MIR132 and RP11-38F22.1 interact with Cathepsin L (CTSL) in the development of IDD. Although the sample size is small in the study, the result indicated that lncRNA contributes to cartilage degeneration to some extent.

Other lncRNAs such as lncRNACIR, AC127391.1, AC128677.4, and IGH are also reported being involved in cartilage-related diseases [15, 16]. These lncRNAs discussed above played certain roles in cartilage development and degeneration and may be the appropriate biomarkers and targets for the treatment of osteoarthritis and intervertebral disc degeneration.

## The role of lncRNAs in cartilage regeneration

Cell, biomaterial, and tissue engineering are the three main approaches for cartilage regeneration. Cell therapies contain transplanting mesenchymal stem cells, autologous chondrocytes, cartilage progenitor cells, and pluripotent stem cells [87]. However, it may take 2–3 years to produce stable and mature ECM after cell transplantation [34]. Biomaterials show simpler regulatory process than cell therapy but do not provide biological function and trigger synthesis of ECM [88, 89]. Tissue engineering combining cells and biomaterials act as a cartilage repair method with unsatisfactory mechanical function [90]. There is still no reliable method to generate articular cartilage to original tissue after injury or disease and no regenerative treatment available for clinical use. Up to now, there is no satisfactory method to cure osteoarthritis and intervertebral disc degeneration.

**Fig. 3:**
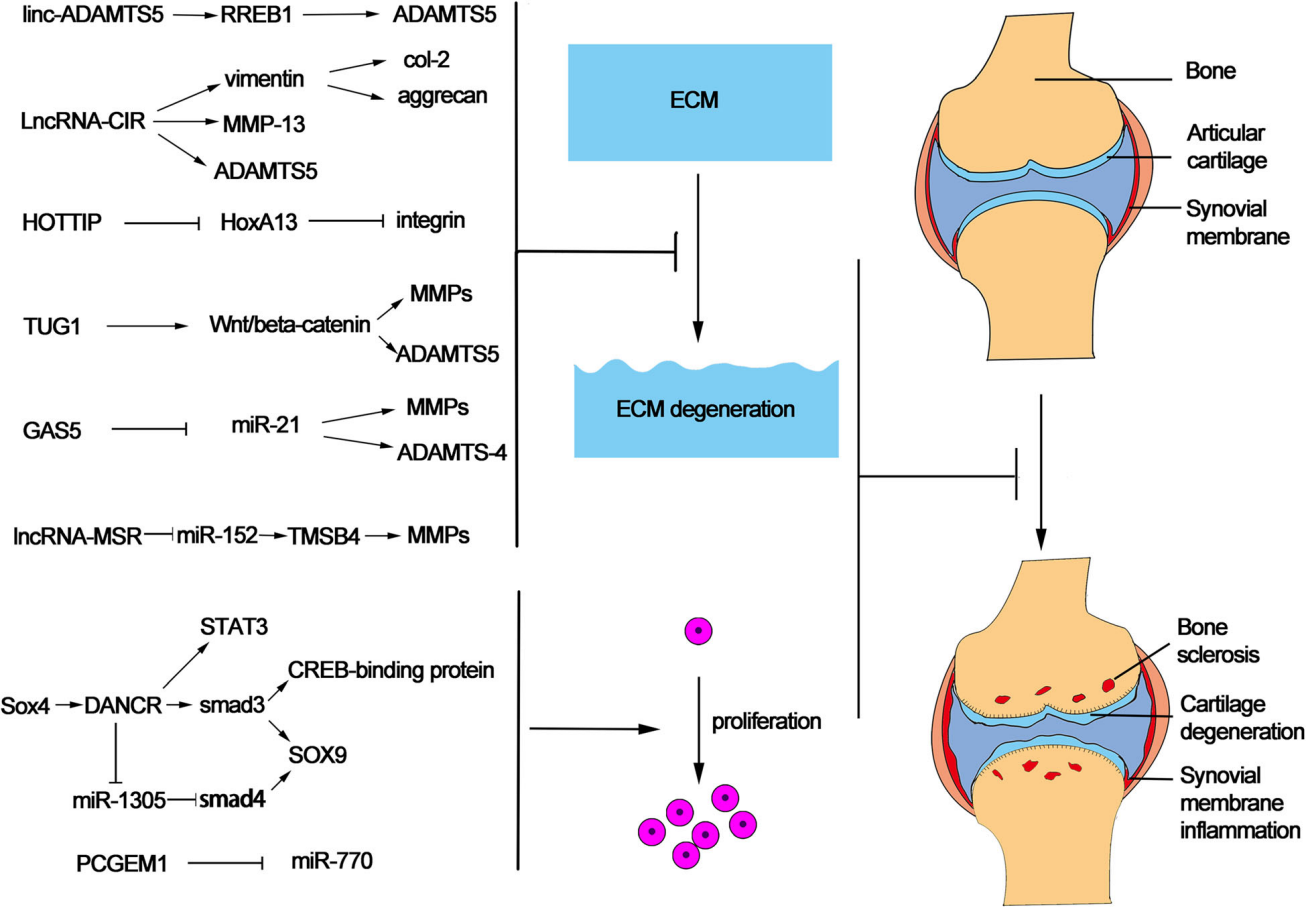
Linc-ADAMTS5, LncRNA-CIR, HOTTIP, TUG1, GAS5, and LncRNA-MSR inhibit the ECM degeneration through influencing the expression of ECM protease MMPs and ADAMTS. Upregulation of lncRNA DANCR and downregulation of PCGEM1 could promote the proliferation of stem cell to regulate the cartilage regeneration

Emerging evidence showed that lncRNAs are involved in cartilage regeneration (Fig. 3). Targeting lncRNA may be a potential method to OA treatment. For example, silencing of HOTAIR could protect against OA development [20]. Liu et al. reported that 82 lncRNAs were upregulated and 70 were downregulated in OA cartilage compared to normal cartilage [15]. LncRNA PTENP1, HOTAIR, HOTTIP, UCA1, TUG1, GAS5, TEA, and EGOA were found to be upregulated, while SNHG4, ncR-uPAR, MERI2C, Emx2os, and DISC were found to be downregulated in the OA chondrocytes compared to normal chondrocytes. It may be a suitable approach to silence the lncRNAs upregulated and overexpress the lncRNAs downregulated in OA cartilage. LncRNA-CIR acts as a siRNA to suppress vimentin whose inhibition results in the reduction of the expression of collagen and aggrecan. Furthermore, lncRNA-CIR was demonstrated as a sponge of miR-27b and could regulate the expression of MMP-13. The study revealed that lncRNA-CIR/miR-27/MMP-13 plays an important role in the degeneration of cartilage ECM [16]. Therefore, silencing lncRNA-CIR may be a potential target for cartilage regeneration. LncRNA growth arrest-specific 5 (GAS5) had been firstly reported as a tumor inhibitor in renal cell carcinoma [91]. GAS5 was also upregulated in osteoarthritis compared to normal tissues [92, 93]. Song and his colleagues [27] found that extraneous GAS5 could upregulate the expressions of MMPs such as MMP-2, MMP-3, MMP-9, MMP-13, and ADAMTS-4. Moreover, LncRNAs could interact with miRNA response elements (MREs) to affect the expression of mRNAs [94]. GAS5 acted as a negative regulator of miR-21 which induced the apoptosis of chondrocytes and cartilage destruction. Therefore, targeting GAS5 may be developed into a novel therapy to OA once its pathophysiology is completely illustrated. Besides, prostate cancer gene expression marker 1 (PCGEM1) was also a possible target for OA therapy. PCGEM1 sponged miR-770 to inhibit the apoptosis of synoviocyte. Exogenous overexpression of PCGEM1 could induce the proliferation of synoviocyte [28]. Another lncRNA HOTTIP is located within the HoxA cluster and worked by interacting with histone modification complexes [95]. The study by Kim et al. [26] illustrated that the expression of HOTTIP was upregulated in OA chondrocytes with modulating HoxA13 gene level. Knocking down HOTTIP expression, a regulator of HoxA gene, would induce chondrogenic differentiation and suppress cartilage degradation. Collectively, although no effective therapies have yet been discovered to stop OA progression, lncRNA may be a potential choice in the future.

Targeting certain lncRNA give a new hope to IDD regeneration. ECM degrading enzymes ADAMTS5 serves as a promoter in IDD development [96, 97]. A newly reported LncRNA named linc-ADAMTS5 is transcribed in the opposite direction to ADAMTS5. Ras-responsive element-binding protein 1 (RREB1) could regulate the expression of ADAMTS5 in NP cells. Linc-ADAMTS5 promotes RREB1 binding to the ADAMTS5 promoter which inhibits the degeneration of ECM [29]. Thus, it was speculated that Linc-ADAMTS5 may play a role in IDD treatment through regulating ECM. Moreover, silencing of lncRNA-CIR was also found to induce the generation of aggrecan and collagen and downregulate the expression of enzymes such as MMP-13 and ADAMTS5 [15]. Chen et al. reported that silencing the expression of lncRNA TUG1 could block the Wnt/beta-catenin pathway to promote NP cell proliferation [30]. When NP cells were transfected with TUG1 siRNA, the Wnt/β-catenin pathway was greatly inhibited with the reduction of apoptosis, but the cell proliferation was obviously enhanced. Thus, silencing lncRNA TUG1 to promote human NP cell proliferation provides a theoretical basis for the clinical treatment of IDD. LncRNA-MSR is a Tymosinβ-4 (TMSB4) pseudogene, which is a subclass of lncRNA. TMSB4 could inhibit the polymerization of actin filament and induce the expression of metalloproteinase in chondrocytes [92]. LncRNA-MSR acts as a ceRNA through competitively binding to miR-152 to upregulate the TMSB4 expression, which leads to the overexpression of metalloproteinase and disorganization of cytoskeleton and degeneration of ECM [31]. LncRNA-MSR may be a potential therapeutic target to decrease the ECM degeneration. The main challenge of these approaches should accomplish with target-specific delivery. Nanoparticle could improve the stability and target-specific of lncRNAs. Delivery of lncRNA with nanoparticle may be a suitable method to be target-specific and deserves further researches. It is clear that a practical approach to silencing a critical lncRNA candidate may have more broad implications than blocking the initiation or progression of osteoarthritis or intervertebral disc disease. For example, silencing MEG3 intensifies lipopolysaccharide-stimulated damage of human lung cells [98]. Downregulation of lncRNA NEAT1 exerts suppressive effects on immunity [99]. Silencing of lncRNA DGCR5 contributes to the growth, migration, and invasion of cervical cancer [100]. Knockdown of lncRNA ROR suppresses proliferation, migration, and angiogenesis in microvascular endothelial cells [101]. Inhibiting lncRNA GAS5 attenuates damage induced by H_2_O_2_ in retinal ganglion cells [102]. Therefore, if we want to target cartilage-related lncRNAs for cartilage regeneration, more attention should be paid to the side effects of the treatment.

Meantime, promoting the expression of lncRNAs which induce chondrogenesis of stem cells may be an attractive approach to cartilage regeneration. Mesenchymal stem cells are an attractive cell source used in cell therapy for degenerative disease. A considerable amount of literatures have described mesenchymal stem cells have the abilities for osteogenic differentiation, adipogenic differentiation, myogenic differentiation, and chondrogenic differentiation [103]. Stem cell transplanting is one of the methods useful for cartilage regeneration. However, how to effectively induce stem cell differentiation into cartilage remains to be explored. Emerging evidences show that lncRNAs play a crucial role in stem cell differentiation. Nguyen and his colleagues found that 230 lncRNAs and 498 associated miRNAs correlated to chondrogenic differentiation [104]. In a microarray test, lncRNA 50450, 37692, and 16667 were prone to promote chondrogenic differentiation of MSCs [105]. MEG3 regulates chondrogenic differentiation of mesenchymal stem cells by inhibiting TRIB2 expression through methyltransferase EZH2-mediated H3K27me3 [106]. H19 acts as a promoter in cartilage differentiation of ADSCs. However, whether STAT2 and IRF9 are the targets of H19 and the underlying mechanism remains to be identified in the following works [107]. LncRNA UCA1 were proved to promote chondrogenic differentiation of human bone marrow mesenchymal stem cells through the TGF-β pathway. miRNA-145-5p/SMAD5 and miRNA-124-3p/SMAD4 axes were the targets of UCA1 in chondrogenic differentiation [108]. LncRNA DANCR was first studied in hepatocellular carcinoma [109]. It plays an important role in chondrogenic differentiation, which is stimulated by Sox4. A survey conducted by Zhang and his colleagues had shown that Sox4 increased the expression of LncRNA DANCR through binding to it to promote the chondrogenesis [32]. Deletion of DANCR reversed the stimulative effect of Sox4 on the chondrogenesis of SMSCs. In addition, the STAT3 pathway was involved in the process of chondrogenic differentiation [110]. Moreover, smad3 could activate the expression of SOX9 and recruit CREB-binding protein to promote chondrogenic differentiation [111]. LncRNA DANCR was also found to induce chondrogenic differentiation through upregulating smad3 and STAT3. Furthermore, overexpression of lncRNA DANCR increased the expression of chondrogenic markers and GAG/DNA ratio [112]. Although lncRNAs are shown as a potential target for cartilage treatment, more evidence about lncRNAs’ successful treatment in vivo should be investigated. These exciting results suggest that targeting lncRNAs or their related signaling pathways might be a feasible approach for cartilage regeneration.

## Conclusions

Only a small number of lncRNAs were fully characterized. Microarray is utilized to detect lncRNAs from a known RNA pool, while RNA sequencing (RNA-seq) could detect new lncRNAs [113]. LncRNAs function through various mechanisms in biological processes [114]. For example, lncRNAs could act as RNA decoy, miRNA sponge, competing endogenous RNAs (ceRNA), RNP components, and chromatin modifier’s recruitment and modulate translation, splicing, and degeneration of mRNA [115–118]. LncRNAs, genes, proteins, and associated pathways are studied in the co-expression network with informatics approach. Numerous technologies were emerging to study lncRNAs such as RNA immunoprecipitation techniques (RIP-Seq), RNA-Seq, crosslinking, and immunoprecipitation followed by high-throughput sequencing (Clip-Seq), chromatin isolation by RNA purification (ChIRP), and capture hybridization analysis of RNA targets (CHART) [119–123]. After that, a loss-of-function experiment should be carried out to verify the function of lncRNAs. SiRNA and shRNA work well to silence RNA but limiting to the cytoplasmic lncRNA [124]. Then, antisense oligonucleotides (ASOs) begin to be used to silence nuclear lncRNAs [125]. Nowadays, clustered regularly interspaced short palindromic repeats (CRISPR/Cas 9) technology is emerging as an ideal tool to knock out the sequence of certain lncRNA [126, 127]. One situation we should bear in mind is that CRISPR/Cas 9 is not suitable for knocking out the lncRNAs located in an exon of other genes. In research, once a novel lncRNA was found, we should illustrate the functions of the lncRNAs and verify the functions in vitro and in vivo. In the process of detecting the expression of lncRNAs between different tissues or species, RNA-seq retains the gold standard to identify novel lncRNAs. Second, structure, subcellular location, and binding partner are the three main approaches to predict the function of lncRNAs [33]. LncRNAs do not have the same sequence conservation as protein-coding genes [128]. As a result, the potential function of lncRNAs cannot be predicted by the sequence. Interestingly, the secondary structure of lncRNAs shows more conservation throughout evolution than sequence [129]. And secondary structure shows more importance in the function of lncRNAs. The function of lncRNAs is widespread, so more attention should be paid to the secondary structure of lncRNAs in the future. Chemical and enzymatic probes are utilized to analyze the second and tertiary structure. And the next-generation methods such as Structure-seq, SHAPE-seq, and FragSeq are emerged useful to analyze the structure of lncRNAs [130–132]. Meantime, the location was related to the function of lncRNAs. To some extent, the function of lncRNAs may be indirectly predicted by the function of genes nearby. Single-molecule RNA in situ hybridization is the prominent method to detect the location of lncRNAs. Moreover, hybridization-based methods are useful to isolate lncRNAs and binding DNA, RNA, and/or protein, which act as a predictor of lncRNA function [133]. In the process of verifying the function of lncRNAs, owing to lncRNAs which show no conservation among species, there are often no homologs of lncRNAs in the animal. It is not easy to find in vivo models to test the function and mechanism of lncRNAs in detail. Nevertheless, many KO animal models for lncRNAs were established with gene disruption, targeted promoter deletions, and premature termination strategies [134–136]. CRISPR-Cas9 is a breakthrough genome editing tool. However, lncRNA may retain the function with CRISPR-Cas9 owing to the lack of ORF [137, 138]. Therefore, the derivative of Cas9 should be developed to remove the entire gene fragments [139, 140]. LncRNA used as an approach to treat cartilage-related disease is in infancy. As reported by Gogtay, nucleic acid could act as a therapeutic agent [95]. Owing to many lncRNAs involved in the pathological process of cartilage-related diseases, lncRNA-targeting therapy may be a new hope for treatment. RNAi, antisense oligonucleotide, locked nucleic acid GapmeRs, small-molecule inhibitors, and zinc finger nucleases are potential choices to silence the cartilage disease-related lncRNAs [61]. Knocking out the lncRNA which induce cartilage degeneration may be useful. Through advancing technologies, knocking out or overexpressing the key lncRNAs may be potential approaches to treat cartilage-related diseases.

## Data Availability

Not applicable

## Abbreviations

OA: Osteoarthritis
IDD: Intervertebral disc degeneration
Sox9: Sex-determining region Y (SRY)-box 9
AHR: Aryl hydrocarbon receptor
BDNF: Brain-derived neurotrophic factor
VEGF: Vascular endothelial growth factor
HOTAIR: Hox transcript antisense intergenic RNA
MMP: Matrix metalloproteinase
PACER: p50-associated cyclooxygenase 2-extragenic RNA
HNPC: Human nucleus pulposus cells
HIF1α: Hypoxia-inducible factor-1α
FAF1: Fas-associated protein factor-1
ROS: Reactive oxygen species
ASOs: Antisense oligonucleotides
CRISPR: Clustered regularly interspaced short palindromic repeats
